# Rare Presentation of Moyamoya Disease with Sub acute Presentation in Iran 

**Published:** 2018

**Authors:** Payam SASANNEJAD, Fateme REZAEI, Reza BIDAKI, Ehsan ZAREPUR

**Affiliations:** 1 Department of Neurology, Mashhad University of Medical Sciences, Mashhad, Iran.; 2 Research Center of Addiction and Behavioral Sciences, Shahid Sadoughi University of Medical Sciences, Yazd , Iran.; 3 Diabetes Research Center, Faculty of Medicine, Shahid Sadoughi University of Medical Sciences, Yazd, Iran.; 4 Student Research Committee, Faculty of Medicine, Shahid Sadoughi University of Medical Sciences, Yazd, Iran.

**Keywords:** Moyamoya disease, Computed Tomography Angiography, Stroke, Magnetic Resonance Imaging

## Abstract

Moyamoya disease is a chronic progressive vascular disease of brain characterized by bilateral stenosis or occlusion of the arteries around the circle of Willis with prominent arterial collateral circulation. We introduce here a patient with Moyamoya who was misdiagnosed. She was a 16-yr-old female from north east of Iran who complained left hemiparesis and was diagnosed Moyamoya disease by brain and cervical CT-Angiography. There was still great difficulty in the diagnosis of diffuse white matter lesions. The CT-Angiography showed bilateral internal carotid stenosis with "puff of smoke" collateralization arising from the circle of Willis, therefore Moyamoya disease was raised. The clinical diagnosis of Moyamoya is challenging and misdiagnosis is probable. Therefore, the physicians should know this disease and think about it in patients with Juvenile stroke. This shows that Moyamoya disease should be considered in differential diagnoses especially among young patients presenting with unexplained cerebrovascular syndromes.

## Introduction

"Moyamoya" is a Japanese term described earlier by Takeuchi and Shimizu and used to describe a pattern like a puff of smoke in the brain imaging (1- 3). However the disease has a particularly high incidence in Eastern Asia, especially in Japan, many further patients have been reported elsewhere, including North America and Europe area. It is a cerebrovascular disease characterized by collateral vascular network in angiography (3, 4). 

Branches, with abnormal vascular networks are in the proximity of the occlusive lesions in arterial phase. It may present as an ischemic or hemorrhagic stroke, which seems later develops more in adults and the former more in children (2, 3). The gold standard diagnostic evaluation of Moyamoya disease is the conventional cerebral angiography, but this method is replaced with other non-invasive neuroimaging like brain Magnetic Resonance Imaging (MRI) and brain Magnetic Resonance Angiography (MRA) (3, 4).

Here, we report a teenager patient with subacute left-sided hemiparesis within 2 weeks first diagnosed as demyelinating disease, and followed deteriorating coarse of illness during her admission. Moyamoya disease, matching with additional imaging, was raised.

## Case presentation

A 16-yr-old female was referred to the Department Neurology Emergency, Qaem Hospital, Mashhad, Iran in 2013, because of left- sided weakness since two weeks ago. Informed consent was taken from the patient. She had a history of an upper respiratory tract infection one week before beginning her symptoms. She had no history for a specific disease and drug use, except some antibiotics for infection mentioned above, including azithromycin 500 mg first and then 250 mg daily up to 5 days. There was not any history of previous trauma. Her symptoms were started and progressed step by step. First, she had left arm pain with developing numbness. The weakness of the left arm and leg was appeared as well as the weakness of the ipsilateral face.

**Fig. 1:**
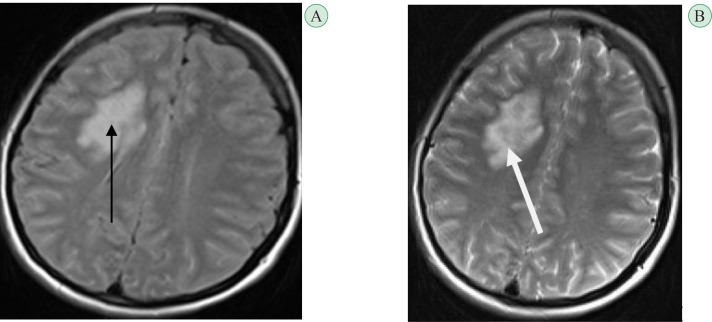
Axial brain MRI of the patient in FLAIR (A) and T2-sequence (B) illustrating involvement of right deep white matter

On neurological examination, she was conscious and oriented. The speech was fluent. Left central facial palsy was seen on her face. Her gait was hemiplegic, muscle tone was increased on the left side of the body, muscle power was 3/5 over left upper and lower extremities, deep tendon reflexes were exaggerated and plantar reflex was upward on the left side. There was no abnormality on her systemic examination including cardiac and pulmonary exam, no fever and no meningeal signs. So, after admission on our hospital, brain MRI was performed. According to the previous history of her upper respiratory tract infection (URTI), the course of onset the symptoms and brain MRI which showed right deep white matter lesions, it was not in accordance with the known vascular territories (Fig. 1). Thereby we initially suspected of a demyelinating process, and she received pulse methylprednisolone 1g/daily for 5 consecutive days. However, other diagnostic measures was carried out at the same time. The laboratory tests consisted of immunologic tests for vasculitis, hematologic (Hyper coagulative tests), and biochemistry tests which were all within normal ranges. 

**Fig. 2:**
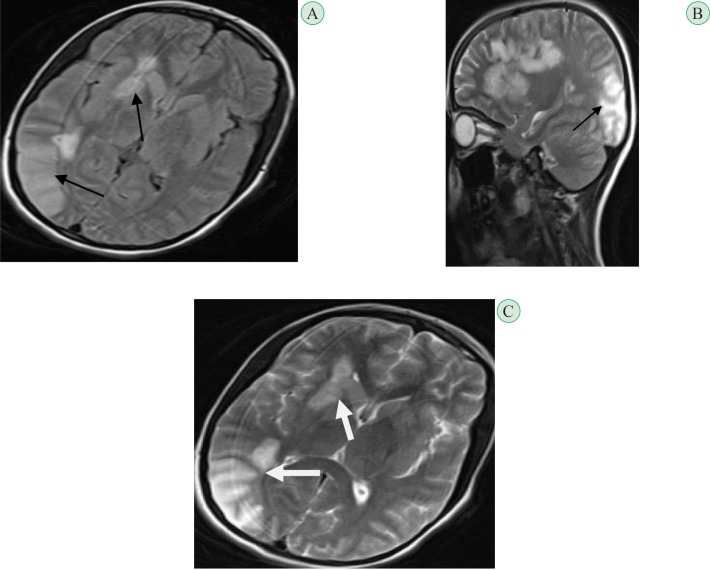
Second brain MRI. **A**. Axial plane, FLAIR sequence **B**. Sagital plane, T2-sequence and **C**. Axial plane, T2-sequenceshowing more developing lesions which seems have a vascular pattern (arrow

**Fig. 3:**
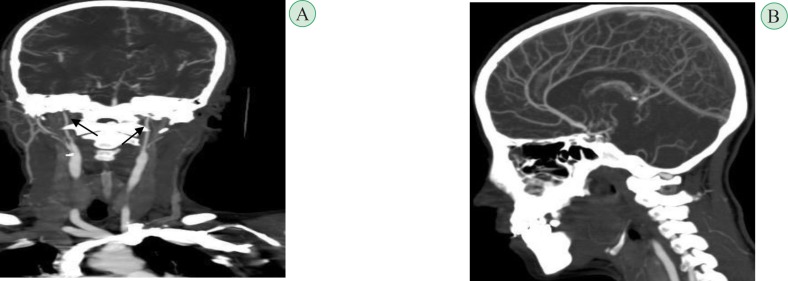
Brain and cervical CT-Angiography. A-Note bilateral narrowed internal carotids (arrows). B-collateral vascular networks appear "puff of smoke" pattern

During her hospitalization, our patient gradually deteriorated. She experienced increasing difficulties with walking and speech as well as progressive hemiparesis to hemiplegia, so additional evaluations were performed. Cerebrospinal fluid analysis was normal. A repeated brain MRI revealed further lesions fitting more on the arterial territories at this time, rather than purely white matter involvement (Fig. 2). For more evaluation of the vascular structure of the brain, brain and cervical CT angiography was done. CT angiography showed bilateral internal carotid stenosis with "puff of smoke" collateralization arising from the circle of Willis, therefore a diagnosis of Moyamoya disease was raised (Fig. 3).

The deteriorating clinical condition and complications of the long-term hospitalization and immobilization, including infections and deep venous thrombosis accompanied with an episode of pulmonary thromboembolism, prevented further evaluation of the patient, including brain angiography. Our patients eventually died as a consequence of the above-mentioned conditions.

## Discussion

The rare diseases may be under diagnosis because of the complex course and rare presentations. The reports about some diseases are very rare about diseases like Moyamoya in Iran. 

Moyamoya disease has a higher incidence in Asian Countries, particularly in Japan (5). There is no valid evidence about its incidence in Iran, but a few cases were reported (6). The evidence show that Moyamoya has two age distribution in two type, ischemic type in the pediatric and hemorrhagic one in the adults (1-5) with a high-frequency in female sex (5). This matter is in concordance with our case.

Heijmen et al. reported a misdiagnosed Moyamoya case as leptomeningeal metastases. A 46 yr- old woman was diagnosed with breast cancer and was treated with immune suppressive agents. She had neurological deficits and after a few years, diagnosis of Moyamoya was raised (5). Khalesi et al. also reported an Iranian case of Moyamoya misdiagnosed as an encephalitis (6). The main clinical features of Moyamoya are some cerebrovascular complications including transient ischemic attack (TIA), ischemic stroke, and seizure. The presentation of the disease could be different depending on the age at the time of diagnosis (5, 6). There is a high-frequency occurrence of the disease in female sex (5). 

Our patient was a young girl without vascular risk factor and lesions on first brain MRI were not on a particular vascular territory. The symptoms were developed sub-acutely, and according to a recent history of upper respiratory tract infection, a demyelinating process was considered initially. However, a few cases of Moyamoya have been reported as subacute presentation such as a 61-yr-old female with a headache and left-sided hemiparesis in whom a subacute subdural hematoma has been found on Computerized Tomography scan and finally Moyamoya disease has been diagnosed (7). 

The etiology of Moyamoya disease is not yet known, however, studies pointed out some factors like a bacterial or viral infection which may play a role (3, 8). A genetic factor may contribute to Moyamoya considering high occurrence among Asian populations (3, 8).

Our Iranian patient was the first child of non-consanguineous parents who did not have the history of the same problem in her family members. However, we could not declare the development of abnormal vasculature in our patient were induced by genetic and ethnicity factor or maybe infectious factor. Kidani et al. reported a case of pure red cell aplasia who received long-term treatment immunosuppressant agents such as prednisolone (1) which could be a rare cause for Moyamoya (1, 8- 11). Our patient had received corticosteroid, first as a mismanagement event. Actually, mismanagement and misdiagnosis of different diseases can result in irreversible consequences (5, 6, 12). 

Our opinion was engaged if latter treatment modality could progress disturbance of vascularity of the brain or deterioration of our patient was an independent process. We could not perform conventional angiography because of the critical general condition of the patient. Unfortunately, the patient was died because of multi infracted lesions.

**In conclusion,** Moyamoya disease should be considered in presenting of neurological deficits in all groups especially in children and young adolescence with the subacute onset and unexplained symptoms related to intracranial ischemia.
